# Society Meetings

**Published:** 1914-10

**Authors:** 


### SOCIETY MEETINGS

---

Brief reports and announcements of meetings of societies of Western New York, and those of general scope, are requested from Secretaries. Copy should be on hand the fifteenth of the month. Full transactions will be published at cost of composition.

---

**The American College of Surgeons.** In view of the animated and sometimes acrimonious comments made on this organization, we publish the list of members in our territory. It seems to us that, on the one hand, the College has admitted men of standing commensurate with its aims and, on the other hand, that the charge of unreasonable restriction of membership is not justified. The College should properly be limited to surgeons, unless in conferring special honor upon a distinguished physician, yet the term surgeon is obviously employed in a broad sense to include various forms of specialization. As the College is still a young organization and has announced a policy of extension of admissions as applicants establish their worthiness, and as many men have undoubtedly been conservative in applying for admission, the charge of exclusiveness is premature.

In one respect, however, we feel like offering a friendly criticism. It is perfectly proper—under proper circumstances—for a member of the profession to announce his affiliations with the various institutions. It occurs to us, however, that the affiliation, in this instance, should be announced in full and not by initials. The initials F. A. C. S., suggest a degree conferred by a legally chartered university or college or by an examining board, such as confers somewhat similar initials in European countries, or at least in Great Britain. With all respect, it may be pointed out that the American College of Physicians is an entirely private organization and, if the initials F. A. C. S. are employed, similar initials, suggesting degrees, may be used with equal propriety by members of other organizations, of still greater exclusiveness. And, we may even expect the reductio ad absurdum of M. A. M. A., M. M. S. S. N. Y., or even H. P. B. A. M.*

Buffalo—A. G. Bennett, C. R. Borzilleri, C. M. Brown, F. J. Carr, M. Clinton, G. F. Cott, J. F. Fairbairn, E. A. Forsyth, L. M. Francis, J. A. Gardner, F. C. Goldsborough, H. E. Hayd, L. Hendee, J. E. King, P. LeBreton, F. P. Lewis, J. H. Lewis, E. P. Lothrop, J. A. MacLeod, M. D. Mann, E. R. McGuire, F. W. McGuire, T. J. McKee, D. C. McKenney, Roswell Park,

---

*High Private, Buffalo Academy of Medicine.

deceased, F. J. Parmenter, W. W. Plummer, W. S. Renner, H. C. Rooth, E. G. Starr, H. R. Trick, N. W. Wilson, T. Wright.

Rochester—E. J. Bissell, R. R. Fitch, C. W. Hennington, E. W. Mulligan, J. O. Roe, J. F. Whitbeck, F. W. Zimmer.

Ithaca—H. B. Besemer, J. S. Kirkendall; Binghampton—A. S. Chittenden, W. A. Moore; Sonyea—G. K. Collier; Jamestown—G. W. Cottis; Utica—T. H. Farrell, J. H. Glass, A. R. Grant; Syracuse—F. H. Flaherty, T. H. Halsted, G. G. Lewis, F. W. Marlow, E. S. Miller, E. S. Van Duyn; Watertown—G. D. Gregor, J. F. McCaw, A. W. Irwin, J. K. Stockwell; Elmira—R. G. Loop; Clifton Springs—J. G. Mumford, S. Robinson; Warsaw—W. R. Thompson.

---

At the Third Annual Meeting of **Alienists and Neurologists** of the U. S. held under the auspices of the Chicago Medical Society, for the purpose of discussing Mental Diseases in their various phases, July 17th, 1914, the committee on "The Causative Forces of Mental Deficiency" reported the following resolutions, which were unanimously adopted: "We feel it unwise at this time to make any recommendations in regard to constructive legislation owing to the lack of proper evaluation of available data as to causes and sources of mental deficiency."

"We do, however, recommend and urge regulation of mental deficients and the furthering of investigations as to the causes and sources."

The committee on the Prevention of Insanity, reported the following resolutions; which were unanimously adopted:

"Whereas, it is well recognized by alienists and neuralogists the world over that certain major factors are the chief causes of physical conditions accompanied by mental derangement and deficiency, and

"Whereas, these major causes are largely, if not wholly, controllable and eradicable, and

"Whereas, these major causes are alcoholism, habit producing drugs, venereal diseases, work in unsanitary and unhygienic surroundings, and hereditary influence including the immigration of the physical and mental unfit.

"Therefore, Be it Resolved, First: That we recommend to the proper state authorities, the absolute control of the sale of alcohol until such time as actual prohibition be enacted.

Second: That the sale of all habit inducing drugs be strictly regulated in all states of the Union.

Third: That municipal or state control of venereal diseases be established, with proper treatment for indigent patients, to the end that the spread of syphilis and gonorrhea be prevented.

Fourth: That proper, special hospitals for the care and treatment of alcoholism and drug addictions be established.

Fifth: That Municipal, state and national inspection of labor conditions be regularly maintained and child labor abolished.

Sixth: That no known defective dangerous to himself and to others, should be permitted to have unrestricted liberty.

Seventh: That adequate teaching of the principals of heredity and sex life be initiated and fostered in the home with the view to its introduction into the curricula of schools—above the grammar grades, this instruction to be given to the sexes separately.

Eighth: That the various states pass reasonable and universal marriage laws, that will be reciprocal, in preventing the marriage of the physical and mental unfit.

Ninth: That a Psychopathic Laboratory be connected with the Criminal Courts, Common Schools, Railroads, Transportation Companies and Public Service Utilities, responsible for the actual safety of the general public should have their employees regularly examined as to their physical and mental fitness.

Tenth: That, inasmuch as state, county and city public health institutions should have as their superintendents, men of highest qualifications, who may devote their best efforts to their tasks, we recommend that all such positions be subject to civil service examinations.

Eleventh: That in addition to the above, we recommend a nation-wide campaign of education conducted through the public press, university and medical schools, boards of health, state, county and city boards of education, women's clubs and other proper educational mediums, upon the true significance of the development—physical, mental and moral—of the individuals and the race and finally, we recommend that a committee be appointed to promote the enactment of the above resolutions.

The committee on "Alcoholism as a Causative Factor of Insanity" reported the following resolutions, which were unanimously adopted:

"Whereas, In the opinion of the meeting of Alienists and Neurologists of the United States in convention assembled, it has been definitely established that alcohol when taken into the system acts as a definite poison to the brain and other tissues; and

"Whereas, The effects of this poison are directly or indirectly responsible for a large proportion of the insane, epileptics, feebleminded, and other forms of mental, moral and physical degeneracy; and

"Whereas, The laws of many states make alcohol freely available for drinking purposes; and therefore cater to the physical, mental and moral degradation of the people; and

"Whereas, Many hospitals for the Insane and other public institutions are now compelled to admit and care for a multitude of inebriates; and

"Whereas, Many states have already established separate colonies for the treatment and re-education of such inebriates, with great benefit to the individuals and to the commonwealths

"Therefore Be it Resolved, That we, unqualifiedly, condemn the use of alcoholic beverages and recommend that the various state legislatures take steps to eliminate such use; and be it further

Resolved, That we recommend the general establishment by all states and territories of special colonies or hospitals for the care of inebriates; and

Resolved, That organized medicine should initiate and carry on a systematic persistent propaganda for the education of the public regarding the deleterious effects of alcohol; and

Be it Further Resolved, That the medical profession should take the lead in securing adequate legislation to the ends herein specified.

The committee on "Syphilis as a Causative Factor of Insanity," reported the following resolutions, which were unanimously adopted:

"Whereas, Syphilis is responsible for a large percentage of all insanity and mental deficiency.

Be It Resolved That:

First: Health Departments, (Municipal and State) should be equipped to make laboratory examinations for Venereal Diseases.

Second: All Hospitals for the Insane should be equipped to make laboratory examinations for Venereal Diseases.

Third: Hospitals and Dispensaries for the treatment of Venereal Diseases, should be provided.

Fourth: Physicians should be compelled by law to report cases of Venereal Diseases, as is now done in other contagious diseases.

Fifth: Applications for marriage should be required to furnish health certificates.

Sixth: Lectures and Bulletins should be offered freely to the public regarding Venereal Diseases.

Seventh: Newspapers should be requested to use their best influence to educate the people concerning Venereal Diseases.

Eight: Sex Hygiene should be taught in the Public Schools, above grammar grades, to the sexes separately.

The proceedings of the Third Annual Meeting of Alienists and Neurologists of the U. S. held under the auspices of the Chicago Medical Society, July 13 to 17, 1914, will be published in one volume by the Illinois State Medical Journal. It will be in double column, the type and size of page the same as the Journal, and will comprise from four to six hundred pages, price $2.00. This book will contain the papers read and their discussions, together with resolutions adopted. The subjects covered are, Acquired Insanity, Epilepsy, Mental Defectives, Alcoholism, Abderhalden Test, Syphilis, etc.

The book will be ready for distribution by October or November, 1914. As only a limited number is left unsubscribed for, those wishing the publication will please send their subscription at once to the Editor of the Illinois State Medical Journal, Dr. Clyde D. Pence, 3338 Ogden Avenue, Chicago, Ill., as there will not be a second edition. The price of the book is $2.00.

---

**The American Association of Obstetricians and Gynaecologists** held its 27th annual meeting in Buffalo, September 15-17, under the presidency of Dr. Charles North Smith of Toledo, Dr. E. Gustave Zinke of Cincinnati being the Secretary, an office formerly held by our esteemed predecessor, the late Lt. Col. W. W. Potter. 60 members were in attendance and about 40 guests. The Buffalo members, Drs. Hayd, Congdon, King and Lothrop, were hosts at luncheons at the Iroquois and the Buffalo Club and the annual banquet was held Wednesday. On Tuesday evening the Association was entertained at the N. Y. State Institute for the Study of Malignant Disease, by Dr. Harvey R. Gaylord and staff, stereopticon and other demonstrations being given of the work of the Institute. The ladies were entertained at luncheon at the Country Club and at the Lake Shore home of Dr. Earl P. Lathrop. Dr. Irving W. Potter of Buffalo was elected to membership. (Note. It is the policy of this Journal not to anticipate, by abstract or otherwise, the publication of scientific proceedings of societies which maintain journals of their own or which publish their transactions in annual volumes, as in the present instance.)

---

**Elmira Academy of Medicine** opened its Fall season on September 2nd. Dr. R. P. Bush read a paper entitled "The Old and New in Medicine."

---

**The Elmira Clinical Society** held its September meeting at the Mineral Spring Hotel at Breezeport, N. Y. The meeting was in the form of an outing. The members drove the eleven miles in their autos, had a chicken dinner and discussed the mineral water.

**The Medical Society of the County of Chemung** held a meeting on September 15th. Program: Local Anaesthesia, Charles Haase; Chronic Ulcer of Stomach, Ross G. Loop.

---

**The Fifth Congress of North American Surgeons** was held at London, Eng., July 27th to August 1st, with an attendance of about 1500. In many respects it was better managed than those of previous years. The admission to all clinics was by ticket, and these were distributed in a very satisfactory manner, thereby avoiding the rush and crowding experienced at the New York and Chicago sessions.

The program committee deserve great credit, having arranged the work in courses so that no two operators covered the same field at the same time, thereby furnishing continuous program for each visitor.

The Congress occurred during vacation time for London Surgeons, yet every man remained on duty and individually and collectively tried to make the week one long to be remembered with pleasure by every American present.

Mr. Lane probably attracted more attention than any one other operator. He gave a clinic each morning and afternoon to a filled amphitheatre. He meets the adverse criticism there to his theory of ''short circuiting'' in the same manner that he did when criticized for his bone work several years ago. He says that nearly all will be converted to his belief in the course of the next ten years. He seems to have plenty of cases to prove all statements. A large amount of energy is being expended by English surgeons to disprove Mr. Lane's conclusions but he quietly points to his records and says ''they speak for themselves.''

The absence of ''American rush'' is apparent and the slower and more deliberate work is often productive of better results. It also allows many operations to be performed by the ''Two-step'' method, thereby reducing the mortality rate. This is made possible because practically all hospital cases are City orders and can be better controlled. The paid operations nearly all go to ''Nurses Homes,'' the name given to private hospitals.

It was interesting to note the progress made by the London surgeons with local anaesthesia. In one hospital 80% of all appendectomy and 50% of all hernia were done under spinal puncture. In all cases shown at the congress tthe puncture was made with patient in dorsal position instead of the sitting posture.

The evening programs were made of unusual interest. In nearly all cases the papers were read by Americans and discussed by Europeans. This gave a broad scope and an opportunity for comparison seldom made possible.

Not to mention Dr. Jones of Liverpool and Dr. Moynihan of Leeds would be to fail to give credit where credit is due. Not only their presence in London, which was an inspiration, but the warm reception extended at their home clinics added very much to the pleasure of the occasion. Many of us felt that Dr. Moynihan's work was equalled by few and surpassed by none.

Ray H. Johnson, M. D., Buffalo, N. Y.

---

**The University of Buffalo** held its opening exercises for all departments September 21. The Freshman Medical Class numbers 30.

---

"**The Travel Study Club of American Physicians,** which made a successful Study Tour of Europe last year, has completed the plans for its 1915 Study Tour to the A. M. A. meeting in San Francisco, Honolulu, Japan, the Philippines, China, with optional return via Siberia and Europe (war permitting) or via Canada. This being the first party of American Physicians ever visiting the Far East and the new possessions of the United States, a most cordial welcome can be expected by authorities and members of the medical profession. The Travel Study Club would like to make its enterprise as representative as possible and asks all those interested to communicate with the Secretary, Dr. Richard Kovas, 236 East 69th Street, New York."

